# Photoswitching between Water‐Tolerant Adhesion and Swift Release by Inverting Liquid Crystal Fingerprint Topography

**DOI:** 10.1002/advs.202004051

**Published:** 2021-02-18

**Authors:** Wei Feng, Liangyong Chu, Matthijn B. de Rooij, Danqing Liu, Dirk J. Broer

**Affiliations:** ^1^ Department of Chemical Engineering and Chemistry Eindhoven University of Technology Den Dolech 2 Eindhoven 5612 AZ The Netherlands; ^2^ Surface Technology and Tribology Faculty of Engineering Technology University of Twente Enschede 7522 NB The Netherlands; ^3^ SCNU‐TUE Joint Lab of Device Integrated Responsive Materials (DIRM) National Center for International Research on Green Optoelectronics South China Normal University Guangzhou 510006 China

**Keywords:** inverting fingerprints, light responsive, liquid crystal polymer networks, surface topography, switchable adhesion

## Abstract

Although switchable adhesive surfaces are important and desirable for soft robotics, it is still challenging to replicate nature's switchable adhesion capability on artificial surfaces, especially for underwater applications. Here polymeric coatings with fingerprint topographies that are capable of switching the surface adhesion upon light illumination are reported. This is achieved via a synergistic combination of surface topographical inversion and spatially selective distribution of adhesive polymers. The surface topographical inversion is accomplished by the anisotropic deformation of the fingerprint‐configured liquid crystal network (LCN) coating upon light‐controlled order parameter modulation. Adhesive and nonadhesive polymers are spatial‐selectively arranged on top of the LCN coating following the alternating homeotropic and planar domains, respectively, where liquid crystal mesogens are orthogonally aligned. The adhesive part is composed of a water‐tolerant adhesive polymer with 3,4‐dihydroxy‐l‐phenylalanine (catechol) groups inspired by mussel byssus. This report presents a dynamic surface with locally alternating nonadhesive indented areas and adhesive elevated areas where the topographical positions can be dynamically changed with light illumination which can serve as smart skins for robotic applications.

## Graphical abstract

Herein, the development of light‐induced switchable adhesive surfaces is presented. They are based on integrating an adhesive catechol polymer with light‐responsive fingerprints in a liquid crystal network. To introduce adhesion, the catechol polymer is applied on the tops of the inverting fingerprints. When fingerprints are reversed, the adhesive polymer is switched to the valley, and is shielded from any surface which is in contact.

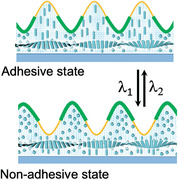

Switchable adhesion is a desirable property for many applications, ranging from the on‐command attachment, and respective detachment, in medical surgeries to robotic locomotion where an adjustable grip promotes advancing.^[^ ^1^, ^2^, ^3^ ^]^ Looking to nature for inspiration, many creatures give exemplary demonstrations. For example, provoked frogs (*Notaden* genus) switch their skins to adhesive by secreting adhesives to deter potential predators.^[^ ^4^, ^5^ ^]^ Geckos,^[^ ^6^, ^7^ ^]^ ants,^[^ ^8^ ^]^ beetles,^[^ ^9^ ^]^ and flies^[^ ^10^ ^]^ have also evolved adaptive adhesive systems assisting to attach/detach from surfaces. In biomimetic studies, scientists developed microstructured surfaces that are suitable for switchable adhesion/detachment in dry environments.^[^ ^11^, ^12^, ^13^, ^14^, ^15^, ^16^ ^]^ Inspired by the byssal thread of mussels, catechol‐group‐containing polymers with impressive adhesive performance have been reported for wet environments.^[^ ^17^, ^18^, ^19^, ^20^ ^]^ However, remotely controllable surfaces with switchable adhesion that can work underwater are still a challenging task, despite impressive progress in artificial adhesives.^[^ ^21^, ^22^, ^23^, ^24^, ^25^, ^26^ ^]^ In particular, coatings functioning assmart skins are desirable in robotics with switchable adhesion/detachment capability underwater without rigid requirement or specific treatment of target surfaces while retaining the advantage of reversible, on‐demand, and fast response.

Here we report a strategy to develop switchable adhesive coatings through synergizing the tunable surface topography and water‐tolerant adhesive into a single functional device. This strategy integrates light‐responsive topographical deformation of liquid crystal network (LCN) coatings with catechol‐group‐containing polymer adhesive spatial selectively distributed on top. The surface of the LCN coating with fingerprint configuration is corrugated, spatially synchronized with the periodicity of chiral helices.^[^ ^27^, ^28^ ^]^ The homeotropic liquid crystal orientations, with the long axes of the molecules on average pointing to the interface, are exclusively in elevated positions, which are controlled with the aid of dichroic dye‐induced material diffusion during polymerization of the LC monomers. On top of the LCN coating, a catechol‐group‐containing adhesive polymer is spatial‐selectively coated, leaving the initially higher parts with adhesive polymer while the lower parts with nonadhesive counterparts. Upon UV illumination, *trans–cis* isomerization of azobenzene moieties incorporated in the LCN induces the order parameter reduction of the LCN. Subsequent opposite topographical responses occur in planar and homeotropic areas: the homeotropic orientation areas contract along the LC director and planar areas expand in the thickness direction, leading to the inversion of the surface topography.^[^ ^29^, ^30^ ^]^ Subsequently, the adhesive homeotropic areas become topographically lower than the nonadhesive planar areas upon UV light stimulation, resulting in the switch of the dynamic coating from adhesive to nonadhesive.

A fingerprint LCN coating with switchable topographical deformation is designed as the base for the functional adhesive. The LCN coating firmly adheres to 3‐(trimethoxysilyl)propyl methacrylate (silane A174)‐coated substrate via covalent bonds, where silane A174 also provides vertical aligning forces for LC monomers prior to polymerization. Together with the twisting forces exerted by the chiral dopant (3*R*,3a*S*,6*S*,6a*S*)‐hexahydrofuro[3,2‐*b*]furan‐3,6‐diyl bis(4‐((4‐(((4‐(acryloyloxy)butoxy)carbonyl)oxy)benzoyl)oxy)benzoate) (compound **6,** in **Figure** **1**A), liquid crystal monomers adopt a fingerprint configuration (Figure 1B, also called “uniform lying helix”) where the helical axes are parallel to the substrate.^[^ ^31^ ^]^ During the light‐induced polymerization of LC monomers, we use 1‐(4‐((4‐((4‐butylphenyl)diazenyl)phenyl)diazenyl)phenyl)pyrrolidine (dichroic dye **4)** to control the relative topographical position of the planar and homeotropic domains (Figure 1C). The dichroic dye **4** shows strong absorption in the wavelength range from 420 to 600 nm with the peak absorption at *λ* = 500 nm (Figure S1, Supporting Information) with a light absorption ratio of 4.84 between planar areas and homeotropic areas. Applying wavelengths of >400 nm for light‐initiated polymerization, the dichroic dye largely suppresses the polymerization in planar areas due to less light penetration, inducing materials to flow from planar to homeotropic areas during the polymerization process, leaving homeotropic regions in topographically higher positions. The height difference between planar and homeotropic orientations after polymerization is ≈400 nm (Figure 1D).^[^ ^32^ ^]^ Notably, the light‐absorption spectrum of dichroic dye **4** does not overlap with the azo crosslinker **1** (((diazene‐1,2‐diylbis(4,1‐phenylene))bis(oxy))bis(propane‐3,1‐diyl) bis(2‐methylacrylate)) (*λ*_max_ = 365 nm) that is added to enable photoswitching, making it possible to use the light of different wavelengths for polymerization and coating stimulation, respectively.

**Figure 1:**
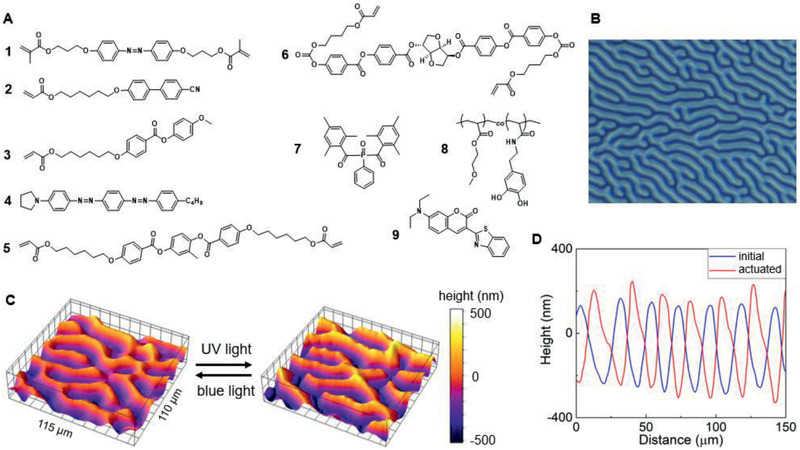
Fabrication of a light‐sensitive fingerprint LCN coating with invertible topography. A) Chemical structures of azobenzene crosslinker **1**; liquid crystal monomers **2**, **3**, and **5**; dichroic dye **4**; chiral dopant **6**; photoinitiator **7**; adhesive polymer **8**; and Coumarin fluorescent dye **9**. B) Image of a fingerprint LCN as observed by polarized optical microscopy. C) 3D view measured by digital holographic microscopy (DHM) showing topographical inversion. D) 2D topographical profiles of the coating surface demonstrating the topographical inversion when actuated by UV light.

Owing to the azobenzene crosslinkers, the order parameter of the light‐sensitive LCN coating can be reduced by the transformation of planar azo *trans*‐isomers to bent *cis*‐isomers.^[^ ^29^ ^]^ Upon reduction of the order parameter, uniaxially aligned LC domains would shrink along with the LC director and expand in the perpendicular direction.^[^ ^33^ ^]^ In the case of present fingerprint LCN coatings, topographical deformation occurs due to different deformation behavior of the alternating areas. Homeotropic areas shrink in the *z*‐direction (thickness direction) and create lateral stress absorbed by neighboring planar areas, contributing to the topographical deformation with planar areas rising. In our experiments, the deformation amplitude is larger than the initial height difference between the two kinds of areas. The surface topography is therefore inversed, as shown in Figure 1C,D. The initially higher parts become lower when stimulated with UV light (365 nm), which can be recovered by the illumination of blue light (455 nm).

The corrugated fingerprint LCN coating is provided with alternating aligned adhesive and nonadhesive parts that follow the fingerprint contour. The fabrication process involves dip coating of adhesive polymer and microcontact printing (MCP) of the nonadhesive layer. Prior to dip coating, the LCN coating was treated with UV–ozone to generate hydroxyl groups on the surface for a firm attachment of the adhesive polymer to the LCN coating.^[^ ^34^ ^]^ The adhesive polymer used here is a catechol‐group‐containing polymer (poly(*N*‐(3,4‐dihydroxyphenethyl)acrylamide‐*co*‐2‐methoxyethyl acrylate (p(DMA‐*co*‐MEA))), polymer **8** in Figure 1A) that is known to be active underwater.^[^ ^17^ ^]^ After the dip‐coating process, the whole LCN coating is coated with a layer of polymer **8** with a thickness of 1 µm, which is measured with an interferometer by comparing the coating thickness change before and after dip coating. Due to the low viscosity of the solution of polymer **8** used in the dip‐coating process, the adhesive layer follows the LCN fingerprint profile. The coating is then illuminated with UV light to induce the planar areas to take higher positions that are used in the following microcontact printing process (**Figure** **2**A). The microcontact printing was conducted with an LC monomer mixture as the ink, which is composed of 42 wt% 6‐((4′‐cyano‐[1,1′‐biphenyl]‐4‐yl)oxy)hexyl acrylate (**2)**, 50 wt% 4‐methoxyphenyl 4‐((6‐(acryloyloxy)hexyl)oxy)benzoate (**3)**, 5 wt% (2‐methyl‐1,4‐phenylene bis(4‐((6‐(acryloyloxy)hexyl)oxy)benzoate)) (**5)**, 2 wt% phenylphosphoryl‐bis(mesitylmethanone) (**7)**, and 1 wt% Coumarin fluorescent dye (**9)**. This coating forms a nonadhesive layer after curing. The presence of the fluorescent dye is for later analysis of the resolution of this process. The relatively high viscosity of the LC ink prevents it from spreading to the whole coating surface, assuring the successful microcontact process with the LC monomers only at the elevated areas. Following polymerization solidifies the ink on top of the coating, and the coating is then illuminated with blue light (455 nm) to convert the azobenzene back into the *trans‐*conformation, making the nonadhesive planar parts dented and converting the coating to the adhesive state.

**Figure 2:**
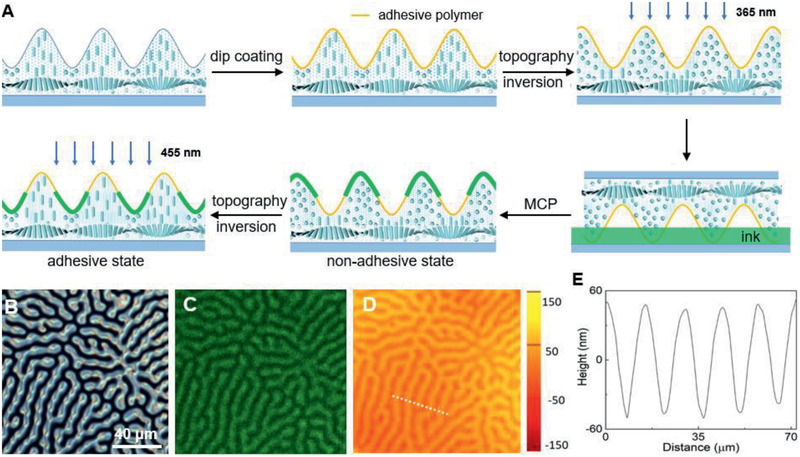
The fabrication process of the switchable adhesive coating. A) Schematic illustration of the dip‐coating and microcontact printing (MCP) processes. The illustration simplifies the molecular alignment. In reality, there is a helicoidal director gradient between planar and homeotropic orientations. B) Microscopy of the fingerprint texture of the region of interest (ROI) as observed between crossed polarizers. C) Correlated confocal fluorescent microscopy of the same ROI, confirming the selective modification of the fingerprint coating with the nonadhesive fluorescent layer. D) Correlated topographic image of the same ROI. E) The surface profile of the cross section is indicated by the white dashed line in panel (D).

The distribution of nonadhesive top coating on the surface is confirmed via correlated polarized microscopy, confocal fluorescent microscopy, and digital holographic microscopy of the same sample area. The birefringent planar areas in the polarized image (Figure 2B) match well with fluorescent areas in the confocal fluorescent image (Figure 2C), while the topographic image shows that the planar areas are lower than homeotropic areas with a height difference of around 100 nm (Figure 2D,E), demonstrating the selective distribution of the fluorescent top coating on planar areas.

With adhesive parts in the higher positions, the not actuated coating is in the adhesive state. The height difference value between two kinds of areas in the not actuated state is smaller (≈100 nm, **Figure** **3**B,C) than that we measure at the pristine LCN coating (300–400 nm, Figure 1C). This is due to the somewhat uneven thickness of the adhesive polymer and the addition of an extra coating on top of initially lower planar areas in the microcontact printing process. The switch from the adhesive to the nonadhesive state is realized through the UV‐induced surface topographical inversion. Initially, the homeotropic areas with the adhesive layer are higher than the nonadhesive planar areas, and the whole coating is adhesive. Upon UV illumination, the *trans–cis* isomerization of the azobenzene crosslinker induces the order parameter reduction of the LCN, resulting in the rise of the planar areas and shrink of the homeotropic areas. As the deformation amplitude is larger than the height difference of two differently treated areas, the surface topography is inverted, with a final height difference value of 350 nm (Figure 3B,C; Movie S1, Supporting Information). In the actuated state, the nonadhesive planar areas are higher than the adhesive homeotropic areas, preventing the contact between the adhesive coating and target objects in practice. Switching from the adhesive to the nonadhesive state proceeds under UV irradiation within 10 s. Switching back can be accelerated by exposure to blue light but takes, depending on light intensity and temperature, somewhat longer (Figure 3D).

**Figure 3:**
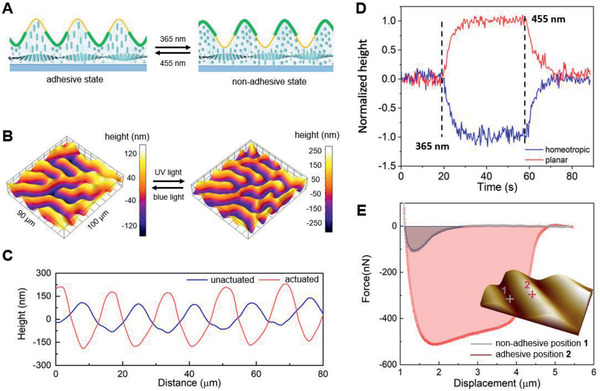
Light‐induced switch of surface adhesion. A) Schematic illustration of the switch of adhesion via light‐triggered topographical deformation. The inversion of the surface topography tunes the adhesive homeotropic areas from protruded to dented, switching the whole coating from an adhesive state to a nonadhesive state. B) 3D images and C) 2D profiles of the surface topographical inversion during the light stimulation process. D) Switching kinetics as probed by displacement at a homeotropic adhesive position and the planar nonadhesive states. E) Force–distance curves in the withdrawal process of a colloidal probe from the adhesive and nonadhesive states within the inset of an AFM image with the two positions.

We measured the localized adhesion by atomic force microscopy (AFM) provided with a colloidal probe under ambient conditions. The retraction forces measured at positions **1** and **2**, as indicated in the inset of Figure 3E, reveal a considerable difference in surface adhesion between different surface areas. From the force curves in Figure 3E, the withdrawing force of the probe distancing from the adhesive areas (525 nN) is 4.8 times larger than from nonadhesive areas (109 nN) in Figure 3E. Notably, the effective displacement range of adhesive force in position **2** is also larger than position **1**, indicating the sticky surface property of position **2**. The local interfacial toughness, represented by the integral area between the force curve and zero force line, indicates a contrast ratio of 20.6 between the nonadhesive position **1** (gray area in Figure 3E) and adhesive position **2** (red area in Figure 3E).

As the adhesive was designed for devices to work under wet conditions, we performed our tests correspondingly. In the first experiment, we measured by AFM the localized adhesive force, marked by the presence or absence of the adhesive, as we described above for ambient conditions. For this purpose, we immersed the coating in water and measured again the displacement force of the colloidal AFM tip, at the two positions. The results are shown in **Figure** **4**A. Here the withdrawing force of the probe distancing from the adhesive areas is 276 nN which is 3.4 times larger than that from nonadhesive areas which are measured to be 82 nN. The difference in work, as calculated from the integrals under the curves, is 4.5 in the underwater conditions. This adhesion contrast, in force values as well in work, is smaller than under ambient conditions, but still of relevance for practical applications.

**Figure 4:**
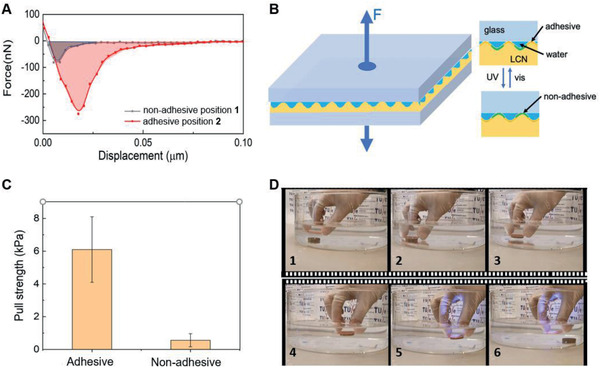
Adhesion tests under wet conditions. A) Force–distance curves in the underwater withdrawal process of a colloidal probe from the nonadhesive (position **1**) and the adhesive position (position **2**). B) Pulling test setup for the mechanical analysis of the switchable adhesion of the dynamic coating surface. C) The contrast of pull strength in the adhesive and nonadhesive states in a wet environment. D) Application of the artificial switchable fingerprint on a glass substrate for the pickup–transport–release of a metallic object. The coating on a glass substrate is used to approach (**1**, **2**), pickup (**3**), and transport (**4**) the copper pellet. Upon arrival at the destination, the adhesive coating becomes nonadhesive upon UV light illumination (**5**) and releases the pellet (**6**).

In the next experiment, the contrast between adhesive and nonadhesive states was quantitatively characterized using a pulling test in a wet environment. The functional coating as applied on a glass substrate (area: *A* ≈ 2 × 2.5 cm^2^) was attached to a testing stage with the coating facing air (Figure 4B). This coating surface was covered with water (≈2 mL) to create a local underwater environment at the active interface of the coating. A clean glass plate bonded with a soft tape on the other side was pressed onto the coating with a pressure of 2 N for 3 min before pulling. Notably, the adhesion between the soft tape and top glass is much stronger than the adhesion between our coating and top glass. The top glass was then pulled up at a speed of 0.2 mm s^−1^ with a computer‐recorded pulling force. The maximum applied force *F* needed to separate the two plates was taken to calculate the adhesive strength *P* using the equation *P* = *F*/*A*. The nonadhesive property was measured similarly with an additional UV exposure (100 mW cm^−2^) for 2 min before applying force to pull up the top glass. The nonadhesive areas stayed long enough in the elevated state to observe a considerably lower pulling strength. The contrast in force between the adhesive state (6.1 kPa) and nonadhesive state (0.56 kPa) is a factor of 10.9 (Figure 4C). The nature of this experiment makes the ridges in the coating fill with water. Upon separation of the adhesive coating from the top plate, capillary bridging might, to some extent, affect the forces measured.

To demonstrate the functionality of the switchable adhesion, we performed adhesion and release experiments of an object immersed in water (Movie S2, Supporting Information). In the initial state, a glass plate adheres to the coating while it detaches upon UV light illumination by switching the surface to nonadhesive. Next, we demonstrated the application of the switchable coating adhesion in a pick‐and‐place experiment, also performed underwater. As shown in Figure 4D and Movie S3 (Supporting Information), when the coating in the adhesive state approaches the metallic pellet (8 g), a 2 N pressure is applied to attach the metallic pellet to the coating. After picking up and transport the pellet to the destination, the UV light (365 nm, 100 mW cm^−2^) is applied to transform the coating into the nonadhesive state. Upon a short exposure (5 s) to UV light, the pellet is released. By illuminating the coating with 455 nm light (≈80 mW cm^−2^), the surface topography is recovered to the nonactuated state, tuning the coating back to the adhesive state. Owing to good thermal conductivity and high heat capacity of water, the photothermal effect underwater is small, and its resulting topographical deformation is negligible. The pickup–transport–release working cycle is demonstrated in Movie S3 (Supporting Information). Notably, in the switchable adhesion demonstration, the pellet is released within 5 s after applying UV illumination. However, the topographical deformation needs ≈10 s to reach the equilibrium (Figure 3D). This mismatch indicates that the switch of surface adhesion is already large enough to manifest a macroscopic effect before the completion of the surface topographical deformation. In the experiments, the weight of the metal pellet, corrected for the buoyancy but ignoring the reduction in contact area due to the fingerprint corrugation, exerts on the interface is 0.22 kPa. The adhesion force is large enough to allow manipulation of the sample by displacing it.

We developed a dynamic coating with tunable surface adhesion that can be switched remotely using light by modulating the contact surface with an object. The switch of adhesion is achieved via the invertible surface topography of a fingerprint LCN coating and a spatially selective administration of catechol‐containing adhesive polymer. The key to constructing the functional switchable adhesive surface is the utilization of dichroic dye to control the initial surface topography and the presence of an azobenzene crosslinker to initiate surface topographical inversion by light actuation. The two switching states are characterized by an adhesion‐on state during which the adhering component is at the tops of the corrugation and an adhesion‐off state during which the adhering component is hidden in the valleys of the corrugation. In contact with an object, this gives a remarkable difference in contact adhesion under dry conditions. But even more remarkably the adhesive switch also retains under wet conditions which we have demonstrated by the pickup and subsequent release of an object immersed in water. This experiment illustrates that the dynamic coating can be used to develop pick‐and‐release transport systems, even when immersed in water. We envision that this approach could be functional on device surfaces to modulate attachment and detachment both in dry and under wet conditions. The process of topographical inversion, which is the basis for the adhesion control, can also be established by other triggers such as temperature or electricity as we have proven in earlier publications.^[^ ^32^ ^]^ When taking the necessary precautions for the combination of electricity and water, it is even feasible to combine adhesion control with wearable electronics.^[^ ^35^ ^]^ An application that is pursued is smart skins for robotic handling, for instance, object manipulation and placement. Also, it opens pathways for soft robots climbing vertical walls. Besides, the switchable adhesive coating would be promising in the removable surgery pads with light as the stimulus to avoid the use of chemicals that may pose damage to the wound.

## Experimental Section

##### Materials

Monomers **1** and **2** were purchased from Synthon Chemicals GmbH & Co. KG. Monomers **3** and **5** were obtained from Merck GmbH. Dichroic dye **4** (commercially identified as G205) was obtained from Hayashibara Biochemical Laboratories, INC., Japan. Chiral dopant **6** was purchased from BASF. Photoinitiator **7** was purchased from Ciba. Adhesive polymer **8** was synthesized according to the procedure described in a previous report.^[^ ^17^ ^]^ Coumarin **6** and silane A174 were obtained from Sigma–Aldrich.

##### Sample Preparation

Adhesive polymer **8** was prepared according to a previous report.^[^ ^17^ ^]^ The fingerprint LCN coating was prepared on silane‐treated glass substrates. The glass substrates were cleaned by ultrasonication in acetone and isopropanol for 10 min, respectively, followed by a UV–ozone treatment for 20 min. Silane A174 solution (1 wt%) in isopropanol/water (v/v = 1:1) solvent was spun to the clean substrate at a speed of 1500 rpm for 30 s, and the substrate was left on a 90 °C hotplate for 30 min for the silanization reaction. The LC monomer mixture (Figure 1A, 7 wt% compound **1**, 40.4 wt% compound **2**, 50 wt% compounds **3**, 1.3 wt% compound **4**, 0.3 wt% compound **6**, and 1 wt% compound **7**) dissolved in dichloromethane with a concentration of 33 w/v% was spun onto the silane‐treated substrate at the speed of 1200 rpm for 30 s. The thin film of the monomer mixture, that had taken the fingerprint configuration, was polymerized using EXFO (light wavelength range 300–600 nm, emission spectrum in Figure S2 in the Supporting Information), and a light filter was used between the light source and samples to allow the transmission of light with wavelength > 400 nm and prevent the isomerization of azobenzene moieties during polymerization. To assure full conversion of the acrylate groups, a two‐step polymerization was carried out: the sample was first illuminated using Ominicure EXFO S2000 with weak light (10 mW cm^−2^ at *λ* = 440 nm) for 10 min and then illuminated using stronger light (50 mW cm^−2^ at *λ* = 440 nm) for 10 min for both sides of the coating, respectively. The sample was then subjected to the postcure at 120 °C for 10 min to fully cure the monomers. The LCN coating was then soaked in ethanol for 1 h to remove the free dichroic dye and dried in a vacuum oven at room temperature.

To modify the LCN coating with spatially alternating distributed adhesive and nonadhesive parts, a dip coating and microcontact printing were conducted. Before the dip coating, the fingerprint LCN coating was treated with UV–ozone for 20 min to slightly oxidize the coating surface for adhesion improvement. To perform dip coating with the adhesive layer, the coating was immersed in the adhesive polymer **8** (Figure 1A) solution (in methanol, 50 mg mL^−1^) at 30 °C for 10 min. The coating was then slowly withdrawn from the adhesive polymer solution at a speed of 5 mm s^−1^. This coating was partly inactivated by applying a thin film of LCN on top of polymer **8** by microcontact printing of nonadhesive parts, and the monomer mixture (42 wt% compound **2**, 50 wt% compound **3**, 5 wt% compound **5**, 2 wt% compound **7**, and 1 wt% compound **9**) was spin‐coated onto a clean silicon wafer to form a thin (≈0.2 µm) ink layer. But before contact printing, the coating must be brought in the reversed fingerprint state by treating it with UV light (365 nm). The fingerprint LCN coating, with the homeotropic areas now in the higher position, was pressed to be in contact with the ink layer for 30 s. Afterward, the LCN coating was detached from the ink and then subjected to another photopolymerization to cure the ink, similar to the one described above for the polymerization of the fingerprints but in the absence of the cut‐off filter. In the last step, the sample was then illuminated with 455 nm light to have the homeotropic areas higher than planar areas.

##### Characterization

Surface topographies were probed with a digital holographic microscope (DHM) (Lyncee Tech). Light‐emitting diode (LED) lamps from Thorlabs were used to emit the monochromatic 365 and 455 nm light. The microcontact printing of the ink with fluorescent dye **9** was confirmed with the confocal laser scanning microscope (CLSM, Leica SP8). The pulling test was performed using a tensile machine EZ‐20 (AmetekTest) with a 100 N load cell.

A Park XE‐100 atomic force microscope from Park Systems was used for surface force mapping and force–distance curve measurements. A FORTA‐10 probe purchased from AppNano was used for surface force mapping using Pinpoint mode AFM. This mode combined height imaging and tip–sample force tracking simultaneously. At each point, the tip performed a cycle of approaching and retracting to determine the tip–sample adhesion force. The maximum force in the approaching phase was set as 10 nN.^[^ ^36^ ^]^ The radius of the tip was about 10 nm and the spring constant of the probe was 1.05 N m^−1^, determined using the thermal noise method.^[^ ^37^ ^]^ For force–distance curve measurements, a colloidal probe purchased from NanoAndMore GmbH was used. The radius of the SiO_2_ sphere on the probe was 5 µm. The spring constant of the probe was 16.20 N m^−1^.

##### Statistical Analysis

Data were expressed as mean ± standard deviation (S.D). Statistical comparisons were made by one‐way one‐way analysis of variance (ANOVA) (for multiple comparisons) followed by Tukey's post‐test: *p < 0.05, **p < 0.01. The topographical deformations in Figure 3D were normalized to clearly show the response kinetics. The area calculations in Figures 3E and 4A were performed using the ImageJ software. Most of other data processing was done with the Origin software. The sample size in Figure 4D for demonstration was 2.5 cm × 2.5 cm.

## Conflict of Interest

The authors declare no conflict of interest.

## Author Contributions

W.F., D.L., and D.J.B. conceived the research. W.F. designed the experiments, prepared samples, investigated the surface topographical deformation, and carried out the demonstrations. L.C. performed the AFM measurements and analyzed the results with M.B.d.R. W.F., D.J.B., and D.L. composed the manuscript with input from all authors.

## Data and Materials Availability Statement

All data needed to evaluate the conclusions in the paper are present in the paper and/or the Supplementary Materials. Additional data related to this paper may be requested from the authors.

## Supporting information

Supporting Information

Supplemental Movie 1

Supplemental Movie 2

Supplemental Movie 3
