# Supplementary material for: Photoswitching between Water‐Tolerant Adhesion and Swift Release by Inverting Liquid Crystal Fingerprint Topography
Source: Adv Sci (Weinh). 2021 Feb 18;8(8):2004051. doi: 10.1002/advs.202004051 (PMC8061410; doi:10.1002/advs.202004051)
Supplement: Supplementary file 1 — Supporting Information [file ADVS-8-2004051-s004.pdf]

© 2020 Wiley-VCH GmbH

ADVANCED SCIENCE
Open Access

Supporting Information

for *Adv. Sci.*, DOI: 10.1002/advs.202004051

Photo-switching between Water-tolerant Adhesion and Swift Release by Inverting Liquid Crystal Fingerprint Topography

Wei Feng,[1] Liangyong Chu,[2] Matthijn B. de Rooij,[2] Danqing Liu,[1,3] Dirk J. Broer [1,3]**

Supplementary Materials

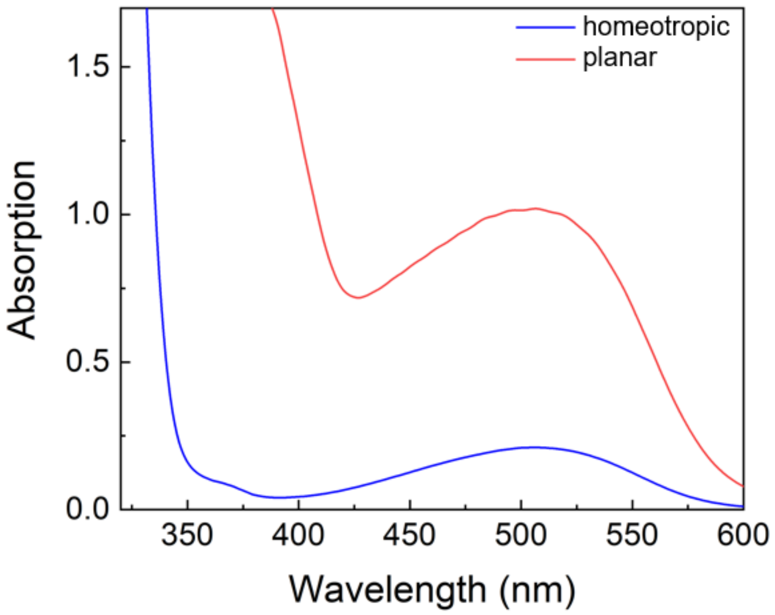

Fig. S1. Light absorption spectra of planar chiral nematic and homeotropic samples with the same concentration of dichroic dye.

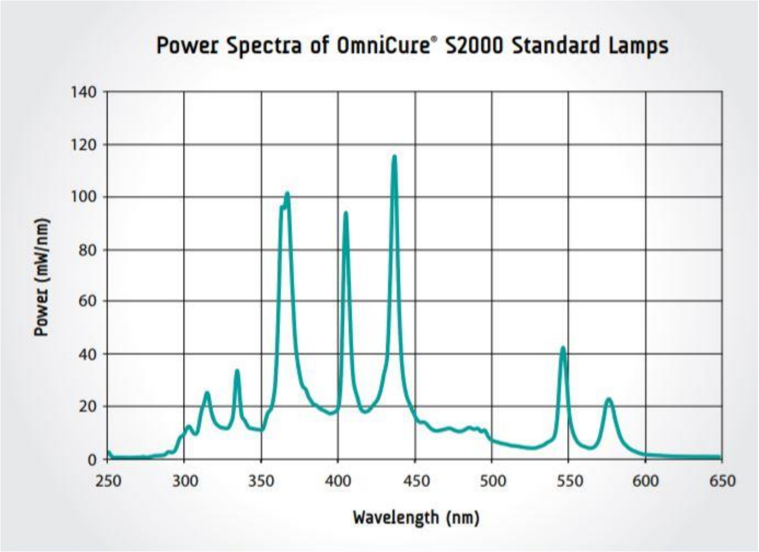

Fig. S2. Spectral output of Ominicure S2000 light source during polymerization. Reproduced from a datasheet provided by the manufacture/distributor.[38]
